# Homicide in the context of psychosis: analysis of prior service utilisation and age at onset of illness and violence

**DOI:** 10.1192/bjo.2023.567

**Published:** 2023-09-19

**Authors:** Stephanie R. Penney, Austin A. Lam, Nathan Kolla, Krystle Martin, Kimberly Belfry, Alexander I. F. Simpson

**Affiliations:** Complex Care and Recovery Program, Forensic Division, Centre for Addiction and Mental Health, Toronto, Ontario, Canada; and Department of Psychiatry, Temerty Faculty of Medicine, University of Toronto, Toronto, Ontario, Canada; Department of Psychiatry, Temerty Faculty of Medicine, University of Toronto, Toronto, Ontario, Canada; Complex Care and Recovery Program, Forensic Division, Centre for Addiction and Mental Health, Toronto, Ontario, Canada; Department of Psychiatry, Temerty Faculty of Medicine, University of Toronto, Toronto, Ontario, Canada; and Waypoint Centre for Mental Health Care, Penetanguishene, Ontario, Canada; Ontario Shores Centre for Mental Health Sciences, Whitby, Ontario, Canada; Waypoint Centre for Mental Health Care, Penetanguishene, Ontario, Canada

**Keywords:** Homicide, psychosis, forensic mental health, service utilisation, victims

## Abstract

**Background:**

Public stigma and fear are heightened in cases of extreme violence perpetrated by persons with serious mental illness (SMI). Prevention efforts require understanding of illness patterns and treatment needs prior to these events unfolding.

**Aims:**

To examine mental health service utilisation by persons who committed homicide and entered into forensic care, to investigate the adequacy of mental healthcare preceding these offences.

**Method:**

Forensic patients across two mental health hospitals in Ontario with an admitting offence of homicide between 2011 and 2021 were identified (*n* = 112). Sociodemographic, clinical and offence-related variables were coded from the health record and reports prepared for the forensic tribunal.

**Results:**

Most patients (75.7%) had mental health contacts preceding the homicide, with 28.4% having a psychiatric in-patient admission in the year prior. For those with service contacts in the year preceding, 50.9% had had only sporadic contact and 70.7% were non-adherent with prescribed medications. Victims were commonly known to the individual (35.7%) and were often family members in care-providing roles (55.4%). Examination of age at onset of illness and offending patterns suggested that most persons admitted to forensic care for homicide act in the context of illness and exhibit a low frequency of pre-homicide offending.

**Conclusions:**

Many individuals admitted to forensic care for homicide have had inadequate mental healthcare leading up to this point. Effective responses to reduce and manage risk should encompass services that proactively address illness-related (e.g. earlier access and better maintenance in care) and criminogenic (e.g. substance use treatment, employment and psychosocial supports) domains.

Available evidence finds that persons with forms of serious mental illness (SMI; typically, psychotic and some major mood disorders) are 4–10 times more likely to commit homicide compared with non-affected members of the general population^1,2^ and that homicides by individuals with psychosis account for approximately 5–10% of all population homicides.^2–4^ Risk for serious violence is elevated during the first episode of psychotic illness, prior to treatment initiation^5^ and in the context of persecutory beliefs.^6^ Furthermore, longer durations of untreated psychosis are associated with a higher proportion of individuals who commit homicide prior to receiving treatment.^7^ At the same time, the contribution of psychosis to the risk of violence is modest in comparison with other criminogenic risk factors, such as criminal history, substance use, unemployment and socioeconomic disadvantage.^8,9^ People with schizophrenia are also nearly twice as likely to be victims than perpetrators of homicide^10,11^ and non-lethal forms of violence.^12,13^

These varying results highlight the need for further research into the precipitants of homicidal violence among persons with SMI and the contributory role of illness. Examining the frequency, adequacy and timing of mental health service utilisation in the period preceding a homicide can shed light on services in need of improvement to reduce rates of these tragic events.^14^

## Mental health service utilisation

Many individuals experiencing mental health problems and who commit homicide are not adequately connected to mental healthcare in the time preceding the offence. Oram and colleagues,^15^ drawing on the National Confidential Inquiry into Suicide and Homicide by People with Mental Illness (NCI) in England and Wales (1997–2008), found that 22.7% of adult domestic homicide perpetrators had been in contact with services in the year before the homicide, including 42.4% of perpetrators who had known symptoms of mental illness at the time of the offence. Individuals who had symptoms of illness at the time of the offence were less likely than perpetrators without symptoms to have previous convictions for violence or a history of alcohol misuse, suggesting that they may be at comparatively lower risk for reoffending despite the severity of violence committed.

Shaw et al,^16^ drawing on the NCI database from 1996 to 1999, found that 12.7% of all individuals convicted of homicide during this time (*n* = 1594) had a lifetime diagnosis of schizophrenia or major affective disorder (e.g. depression, depression with psychotic features), and this increased to 34.2% when including diagnoses of personality and substance use disorders. Ten per cent (10.3%) were judged to exhibit active symptoms of illness at the time of the homicide and 9.1% had had contact with mental health services within 12 months of the offence. Most perpetrators with a history of mental disorder were not acutely ill when they offended and most had never received mental healthcare. Focusing on the subset of offenders with schizophrenia in this sample (*n* = 85), Meehan et al^17^ reported that 50.6% of these individuals were in contact with mental health services in the year preceding the offence, but had elevated rates of non-adherence and missed appointments in the month prior. Two-thirds (68.2%) of these individuals were symptomatic at the time of the offence. Recently, Baird et al^18^ expanded this sample through to 2012 (*n* = 160). When compared with a matched sample of men with schizophrenia and without homicide offences (*n* = 542), those convicted of homicide were more likely to have histories of violence, as well as comorbid personality and substance use disorders. They were also more likely to have missed their last contact with services prior to the offence and to have been non-adherent with their treatment plan.

Having examined all homicides in New Zealand between 1970 and 2000, Simpson et al^19^ found that 8.7% (130 of 1498) could be classified as mentally abnormal based on legal definitions (e.g. adjudicated not guilty by reason of insanity (NGRI)). Of this sample, 58.7% had a psychotic disorder and 51.6% had one or more previous psychiatric hospital admissions (lifetime). One-fifth (19.8%) of these admissions occurred within the year preceding the homicide. Conversely, 29.4% of persons committing ‘mentally abnormal homicide’ had no prior psychiatric contact.

Individuals who commit homicide and who are adjudged to meet legal criteria for a diminished responsibility defence will comprise a distinct group in terms of the contributory role of illness (most often psychosis) in the commission of the offence. The preventive role of mental healthcare in the time preceding the homicide will also probably be clearer in this group and show greater potential for harm reduction. At present, there are few reliable estimates of the frequency, timing, and adequacy of mental healthcare received in this population, where services could have played a key role in averting some of these events.

## Age at onset of illness and violence

Previous research has identified different developmental trajectories of individuals with SMI who engage in violence and antisocial behaviour.^20–23^ A distinction is commonly made between those for whom the onset of violence and antisociality occurs prior to the onset of illness, often in childhood or early adolescence (‘early starters’), and those for whom violence/antisociality emerges contemporaneously with or subsequent to the onset of SMI (‘late starters’). It has also been proposed that a subset of late starters have no history of violence or antisocial conduct, but have chronic symptoms of SMI and commit a single and serious violent offence long after illness onset (‘late late starters’).^21,23,24^ Victims in these cases are often family members or caregivers, consistent with studies reporting high rates of familial victims among homicide offenders with schizophrenia.^25,26^

Examining age at onset typologies can help clarify pathways and risk factors leading to violence among individuals with SMI and target intervention strategies appropriately.^20^ Individuals who begin offending earlier in life, before the onset of illness, are thought to be affected more by criminogenic risk factors (e.g. substance misuse, exposure to violence, personality dysfunction), whereas late starters exhibit violent behaviour primarily in the context of mental illness (see^23^ for a synthesis of studies). Presumably then, risk management strategies would need to differ accordingly to be maximally effective,^27^ and efforts to provide better, earlier and more accessible mental healthcare may be differentially effective in the late-starter groups. Examining the prevalence of severe or lethal violence across early and late starters, as well as differences in victim type (e.g. family members, strangers), can help to clarify risk issues and optimise prevention efforts.

## The current study

Despite growing research on the prevalence of homicides committed by persons with SMI, comparatively less is known about the antecedents of these events and, in particular, the frequency and sufficiency of mental health services received. An examination of service use preceding episodes of serious violence will permit a more precise identification of missed opportunities to provide treatment that could, in turn, reduce risk and potentially avert some incidents of violence. Among groups where the contributory role of illness to homicide is clearer (e.g. persons meeting legal criteria for a diminished responsibility defence), further work is needed to describe specific characteristics of the homicide, such as its timing in relation to illness onset and victim type, to better define risk domains and tailor treatment effectively.

In this context, we investigated mental health service utilisation in a sample of forensic patients who had committed homicide to better understand the adequacy of mental healthcare preceding these offences. We described patterns of age at onset of illness and offending in this group to assess whether findings emerge similar to those in the broader literature on age at onset typologies of persons with SMI who offend. Here, we compared the prevalence of early-starter and late-starter groups in this sample of patients who had committed homicide to the prevalence in all prior studies (*n* = 9) reporting on the prevalence of early starters and late starters among forensic service users. Last, we explored differences across the age at onset groups on a subset of variables focused on prior mental health service utilisation, prior criminal justice contacts, current substance use and personality disorder comorbidities, as well as victim type for the current homicide offence.

Consistent with prior theory^21^ and empirical work (e.g.^23,24^), we hypothesised that the current sample would be characterised by a high proportion of late starters with family victims, and a comparatively lower rate of criminogenic risk factors such as prior criminal justice contacts, substance use and personality disorder. We further expected that a substantial number of patients in this sample will have had gaps in mental health service engagement in the year preceding the homicide offence.

## Method

### Study design and participants

Data were drawn from the forensic patient population across three mental health hospitals in Ontario, Canada. Each hospital's respective forensic programme serves a similar patient population, representing persons adjudicated unfit to stand trial (UST) or not criminally responsible on account of mental disorder (NCRMD; NGRI in other jurisdictions) in the province of Ontario. Section 16 of the Canadian Criminal Code^28^ encompasses the legal principle that, if certain criteria are met, no person who was suffering from a mental disorder at the time of the commission of an offence may be convicted of a crime. The legal test requires that the mental disorder render the defendant incapable of (a) appreciating the nature or consequences of his or her actions, (b) knowing the legal or moral wrongfulness of the offence and/or (c) applying this knowledge in a rational way to the alleged criminal act. In Ontario, over the past decade, there have been an average of 200 new UST and NCRMD cases per year, paired with approximately 125 absolute discharges annually. In 2020–2021 (the latest available data) there were approximately 1700 forensic mental health patients in Ontario.^29^

All forensic patients in Canada are managed under the legal oversight of provincial review boards, which annually review the status of every person under its jurisdiction. For each annual hearing, a psychiatric report is provided and the Ontario Review Board hears evidence and produces a Reasons for Disposition document. We relied primarily on these two documents (appearing in the patient record) to code the variables used in this study. The main points of difference between the three hospital settings pertain to security level (one hospital comprises the province's only maximum security programme and is for men only, whereas the other two include medium and minimum secure services) and catchment area (one urban, one suburban and one rural setting).

All forensic patients adjudicated NCRMD for an index offence of (or including) homicide and who were active in-patients or out-patients between 2011 and 2021 were identified (*n* = 112). Patients were primarily male (93.8%), with a mean age of 35.34 years (s.d. = 12.13) at the time of admission to forensic care. The most frequent primary diagnosis was schizophrenia (80.7, and 94.5% with any psychotic disorder), with half (50.0%) diagnosed with a comorbid substance use disorder. Personality disorders were present in 28.6% (most commonly, antisocial or borderline). Mood disorders were infrequent, with 6.3% of the sample diagnosed with bipolar disorder (*n* = 3) or major depression (*n* = 4). Three individuals (2.8%) were homeless at the time of the index offence. Half (48.4%) had prior criminal convictions preceding the homicide offence for which they were forensically dispositioned.

### Procedure and measures

A coding scheme was developed containing all clinical and risk-related variables investigated in this study. All variables were coded from the health record and relied primarily on the psychiatric reports and the Ontario Review Board's Reasons for Disposition documents described above. These reports are comprehensive and are informed by both collateral (e.g. family, police) and professional (e.g. previous and current treatment providers) sources. Coding was carried out by trained research analysts at each hospital site. Interrater reliability was demonstrated between these analysts and each of two senior researchers for all variables (single-rater intraclass correlation coefficient, absolute agreement, two-way random effects model ≥0.75; kappa coefficient for categorical variables).

Operational definitions of the study variables appear below. Owing to the archival nature of the data, direct patient consent was not required. The study was approved by each hospital's institutional ethics review board (Centre for Addiction and Mental Health REB 155-2018, Ontario Shores REB 20-001-D, Waypoint CRRA#21.06.29) prior to the commencement of data collection. All procedures contributing to this work complied with the ethical standards of the relevant national and institutional committees on human experimentation and with the Helsinki Declaration of 1975, as revised in 2008.

#### Age at onset of psychiatric symptoms

This was based on self- and/or collateral report. Symptoms included positive symptoms of psychosis (delusions, hallucinations, thought or behavioural disorganisation) and mood disorder symptoms such as depression, mania or anxiety. Symptoms of personality and substance use disorders were not included here. We also coded the person's age when they were first prescribed medication for a psychotic or major mood disorder; prescriptions could be made by a psychiatrist or family physician.

#### Age at first hospital admission for psychiatric reasons

This variable included general hospital, civil psychiatric or forensic admissions, and encompassed emergency, short-term or long-stay admissions. We also coded the age at first contact with mental health services, defined broadly to include contact with a mental health professional (e.g. psychiatrist, psychologist, social worker, addictions counsellor) for any reason prior to the index offence.

#### Age at first arrest, number of previous charges and convictions

This information is detailed in the psychiatric reports and obtained through the Canadian Police Information Centre (CPIC). Information pertaining to juvenile arrests and convictions is not typically included in CPIC reports, so this information was based on self-report and/or collateral information.

#### Index offence

This included the index criminal offence(s) for which the patient was found NCRMD. Included offences were first and second degree murder, manslaughter and criminal negligence causing death. Victims of the homicide were categorised as: family member, friend, acquaintance (e.g. co-worker, co-patient), neighbour/roommate or stranger.

#### Mental health service utilisation in the 12 months preceding the index offence

We counted this as present when there was evidence of an ongoing relationship with a professional providing mental health treatment (i.e. two or more visits) and it included a hospital admission for psychiatric reasons. When this was present, we quantified the frequency of service utilisation over the 12-month period as: (a) infrequent (four visits or fewer); (b) slightly frequent (five to eight visits); (c) fairly frequent (less than once per month); (d) frequent (around once per month); and (e) very frequent (more than once per month). If someone did not have quantifiable mentions of mental health contacts in their file but did have mention of regular participation in a mental health-related programme or service we classified this as slightly or fairly frequent (rather than infrequent). In general, we used the numerical frequency of contacts to code this variable, but also took into consideration non-quantifiable mental health contacts/relationships where it was clear that there was more than infrequent contact.

#### Medication use and adherence

We coded whether the individual was being prescribed psychotropic medication covering the time of the index offence and, if so, whether there was evidence of adherence or a lack thereof. Judgements of adherence were based on the patient's self-report and/or corroborating information (e.g. results from blood or urine screens, or reports/suspicions of non-adherence from service providers or family members based on re-emergence of symptoms). If the individual was noted to have partial adherence to such a degree that medication would be unlikely to be effective, this was scored as non-adherent.

### Data analysis

We calculated descriptive statistics to examine rates of mental health service utilisation in the 12 months preceding the index homicide offence, as well as over the person's lifetime. This included any previous pharmacotherapy as well as previous contact with the forensic mental health system. As described above, we coded the frequency of service utilisation for those individuals with contacts occurring in the year preceding the homicide event. Chi-squared tests and one-way analyses of variance were used to compare patients with and without prior mental health contacts on specified clinical (e.g. age, duration of illness prior to offence, rates of substance use and personality disorder diagnoses) and legal/offence-related variables (e.g. rates of prior criminal involvement, victim type). Parallel analyses were conducted across the early- and late-starter age at onset groups, operationalised below. Four variables had more than 10% of cases missing: presence of any prior in-patient admission (cases available: *n* = 89), any admission in the 12 months preceding the homicide offence (*n* = 74), any prior compulsory treatment orders (*n* = 73) and any prior criminal justice contacts (*n* = 82); all remaining variables had less missing data than this. All percentages reported below represent the valid percentage using all cases available and not the percentage that would be obtained using the full sample size (*N* = 112) as the denominator.

#### Subgroup classification

Early starters were defined as patients for whom the age at which they first experienced psychiatric symptoms was greater than the age when they were first arrested or charged with a criminal offence (*n* = 35; 31.3%). Late starters were defined as those whose age at emergence of first known psychiatric symptoms was less than or equal to the age when they were first arrested or charged with a criminal offence (*n* = 77; 68.7%).

The late-starter group was subdivided into two subgroups: those who had suffered fewer than 10 years of illness before their first arrest/charge and who were younger than 37 years at the time of their first offence (late starters; *n* = 67) and those who had experienced 10 or more years of illness before their first arrest/charge and who were 37 years or older when they were first arrested (late late starters; *n* = 10). The duration of 10 years was used as a proxy for chronic mental illness and was the 75th percentile of illness duration within the group. The age of 37 years was used to align with Hodgins,^21^ who described late late starters as typically being in their late 30s or early 40s.

## Results

### Previous mental health service utilisation (lifetime)

Sixty-six individuals (74.2%) had had one or more psychiatric hospital admissions at any point preceding the index offence. Conversely, for 27 individuals (24.3%), their first mental health contact was at the point of forensic admission for the homicide offence. This latter group of individuals tended to be younger at the time of the homicide (mean 28.33, s.d. = 13.48, versus mean 33.94, s.d. = 11.28 for those with prior mental health contacts; *F*_1,109_ = 4.58, *η*^2^ = 0.04, *P* = 0.04). They were also observed to have had a shorter duration of illness (measured in years) preceding the offence and subsequent forensic admission (mean 2.85, s.d. = 4.31, versus mean 0.03, s.d. = 9.15 for those with prior mental health contacts; *F*_1,95_ = 11.57, *η*^2^ = 0.11, *P* < 0.001). When compared with individuals without any previous mental health service utilisation, those with prior contacts had a higher likelihood of having previous arrests or criminal charges (χ^2^(1, *n* = 76) = 5.01, Cramer's *V* = 0.26, *P* = 0.03). There were no differences in the prevalence of substance use or personality disorder diagnoses across those with and without previous mental health service utilisation.

Individuals in this sample had had symptoms of SMI for an average of 8 years at the time of the index offence (mean 8.55 years, s.d. = 8.86, range 0–36) and 30.7% (*n* = 32) had symptom onset and the index offence occur within 2 years of each other. An average of 3 years had elapsed between symptom onset and when the person first received psychotropic medication (mean 3.26 years, s.d. = 5.21, range 0–26). Half of individuals (53.7%) received pharmacotherapy within a year of symptom onset. Thirty-two individuals (43.8%) had a history of prior compulsory treatment orders (e.g. being detained under the Ontario Mental Health Act). No individuals had a previous tenure under the forensic system (Ontario Review Board) for separate offences prior to the homicide offence.

### Mental health service utilisation in the 12 months preceding the index offence

Fifty-one individuals (48.1%) had a documented relationship with a mental health service provider in the year preceding the index homicide offence, including 28.4% (*n* = 21) with an in-patient admission. Of those individuals with any type of mental health contact in the year preceding the offence, 50.9% were having infrequent visits throughout the year, 21.8% slightly frequent, 14.6% fairly frequent, 7.3% frequent and 5.5% very frequent.

Forty-one individuals (38.3%) were prescribed psychotropic medication covering the time of the index offence. However, of those with an active prescription, just 29.3% were judged to be adherent. Fourteen individuals (12.5%) were prescribed clozapine covering the time of the index offence, suggesting that there had been prior problems with treatment responsiveness and symptom improvement (hence the initiation of clozapine).

### Victim type

The majority (55.4%) of individuals in this sample had offended against a family member. Victims in these cases were most often a parent, followed by a spouse. Victims of the homicide offence were friends or acquaintances in 17.9% of cases, followed by neighbours/roommates (17.9%) and strangers (8.9%).

### Age at onset of psychiatric symptoms

As noted, 31.3% of the sample was classified as early starters, 59.8% as late starters and 8.9% as late late starters. Table 1 demonstrates that these obtained frequencies closely resemble those of Laajasalo & Hakkanen^30^ as well as Sánchez-SanSegundo et al^31^ – two studies that included high proportions of patients with homicide offences – as well as Tengström et al.^32^ The remaining studies in Table 1 found higher proportions of early starters and correspondingly lower proportions of late starters. Our observation of a small group of late-late-starter offenders aligns with a comparably sized group found by both Simpson et al^23^ and van Dongen et al.^24^ To our knowledge these are the only two studies that have investigated the late-late-starter group.

**Table 1:** Prevalence of early- and late-starter groups^a^ in prior studies of forensic patients

		Prevalence, %		
Study	Classification of groups	Homicide	Early starters	Late starters
Crocker et al (2018)^20^^b^ – 1800 adults found NCR in British Columbia (*n* = 222), Quebec (*n* = 1094) and Ontario (*n* = 484) between 2000 and 2005	First criminal charge before (ES) or after (LS) first contact with mental health services	6.8	32.2	52.4
Jones et al (2010)^46^ – 1594 first admissions with a diagnosis of schizophrenia to one of three high security hospitals in the UK between 1972 and 2000	ES: those with ≥1 court appearance before first ever psychiatric admission; LS: those with a court appearance after first psychiatric admission	n.r.	53.5	46.5
Laajasalo & Hakkanen (2005)^30^ – 109 Finnish persons with schizophrenia accused of a homicide between 1983 and 2002	ES: convicted of a crime at the age of 18 or before; LS: convicted of a crime after age 18	100	25.0	75.0
Mathieu & Côté (2009)^51^ – 137 men with psychotic or major affective disorders and sentenced to prison (*n* = 44), found NCR (*n* = 59) or involuntary in-patients (*n* = 34) in Quebec (1998–2007)	Presence (ES) or absence (LS) of a conduct disorder diagnosis before age 15	n.r.	59.1	40.9
Pedersen et al (2010)^52^ – 78 men with schizophrenia discharged from a forensic hospital in Denmark in 2001–2002	Presence (ES) or absence (LS) of antisocial behaviour before age 18 (≥1 on item 11 of the PCL:SV)	n.r.	52.6	47.4
Sánchez-SanSegundo et al (2014)^31^ – 88 men with psychotic or major affective disorders found NCR for one or more violent offences in Spain	Presence (ES) or absence (LS) of a conduct disorder diagnosis before age 15	42.1	25.0	75.0
Simpson et al (2015)^23^^c^ – 232 adults found NCR in Ontario between 2002 and 2012, all with psychosis and a history of violent offending	Age at first arrest < (ES) or ≥ (LS) age at first psychiatric symptoms; LLS: same as LS but with ≥10 years of illness duration and whose offending started after age 37	10.4	25.4	66.4 (LS) 8.2 (LLS)
Tengström et al (2001)^32^ – 272 men with schizophrenia undergoing a pretrial psychiatric assessment for a violent offence in Sweden between 1988 and 1995	ES: convicted of a crime at the age of 18 or before; LS: convicted of a crime after age 18	13.0	26.8	73.2
van Dongen et al (2014)^24^ – 223 patient records drawn from the Netherlands Institute of Forensic Psychiatry and Psychology archives, 1993–2008; all patients had a history of schizophrenia and criminal offending	ES: offending started before the onset of illness; LS: offending started after the onset of illness and age ≤34 years at the time of first offence; LLS: same as LS but offending started at age ≥35 years	52.9	43.5	44.8 (LS) 11.7 (LLS)

NCR, not criminally responsible; n.r., not reported. PCL:SV, Psychopathy Checklist, Screening Version.

a. Early starters (ES), late starters (LS) and late late starters (LLS) as defined in the respective studies.

b. This study also included a group of ‘first presenters’, defined as simultaneous onset of illness and offending (15.4%).

c. Twenty-four individuals in this sample overlap with the current sample.

Group differences in prior mental health service utilisation, prior criminal justice contacts, comorbid substance use and personality disorders, as well as victim type for the homicide offence are presented in Table 2. As expected, the early-starter group was found to be younger at the onset of offending compared with the late-starter and late-late-starter groups. The early-starter group also had significantly more prior arrests/charges than the other groups and were more often diagnosed with substance use and personality disorders. The late-starter and late-late-starter groups were found to have a higher proportion of family member victims and fewer acquaintance and stranger victims (late late starters had no stranger victims), compared with the early-starter group. The overall model statistic in these latter cases trended to significance (*P* = 0.07 and *P* = 0.10 for acquaintance and stranger victims respectively), probably reflecting the small cell sizes when the sample was partitioned in this manner. There were no observed group differences in the proportion of individuals with prior psychiatric admissions (lifetime), nor in the frequency of mental health contacts in the 12 months prior to the index offence.

**Table 2:** Differences in prior mental health and criminal justice contacts, diagnosis and victim type across age at onset groups^a^

	Early starters (*n* = 35)	Late starters (*n* = 67)	Late late starters (*n* = 10)	*F* or χ^2^ (*P*)
Age at onset of SMI, years: mean (s.d.)	23.85 (10.82)	25.56 (13.07)	21.22 (7.82)	0.62 (0.54)
Age at onset of offending, years: mean (s.d.)	20.60 (7.99)^b^	29.22 (11.99)^c^	40.60 (5.34)^d^	16.38 (<0.001)
% with prior psychiatric admissions	61.5	77.4	90.0	3.75 (0.15)
% with prior criminal justice contacts (arrests or charges)	97.0^b^	50.0^c^	55.6^c^	19.75 (<0.001)
% with substance use disorder	71.4^b^	41.8^c^	30.0^c^	9.84 (0.01)
% with personality disorder	51.4^b^	16.4^c^	30.0^c^	13.82 (<0.001)
Victim type, %				
Family member	37.1^b^	61.2^c^	80.0^c^	8.08 (0.02)
Friend/acquaintance	28.6	14.9	0.0	5.31 (0.07)
Neighbour/roommate	17.1	17.9	20.0	0.04 (0.98)
Stranger	17.1	6.0	0.0	4.61 (0.10)
Nature of mental health contact – 12 months prior to index offence				
Clear/ongoing relationship, %	40.6	53.1	40.0	1.12 (0.57)
No contact, %	56.3	42.2	60.0	2.32 (0.31)
Infrequent contact, %	28.1	29.7	0.0	3.99 (0.14)
Medications prescribed, %	34.4	36.9	60.0	2.20 (0.33)

SMI, serious mental illness.

a. Early starters: age at onset of illness greater than age at first criminal offence; late starters: age at onset of illness less than or equal to age at first criminal offence; late late starters: ≥10 years of illness before first criminal offence and ≥37 years when first arrested. The sample sizes for prior psychiatric admissions and criminal justice contacts are 89 and 82 respectively, owing to missing data.

^b, c, d.^Values in the same row that do not share subscripts differ at *P* ≤ 0.05.

## Discussion

### Missed preventive opportunities

There is a paucity of research on the timing and utilisation of mental health services prior to the commission of homicide among individuals with SMI. To the extent that some of these homicides occur in the context of first episodes of psychosis or suboptimally treated psychosis, the provision of earlier and more intensive services could have significant harm reduction potential. Still, it is notable that improvements to the quality and availability of community mental health services in many industrialised nations after deinstitutionalisation have not affected the rate of SMI-associated homicide as would have been expected.^14,19,33–35^ Similarly, the more widespread availability of community care and first-episode services would have been expected to reduce a substantial proportion of homicides related to first-episode events. This has also not materialised.^36,37^ At present, population-based data are required to better link the effects of these early and more assertive community interventions to homicide rates among those with SMI. Systems- and other macro-level variables (e.g. the relative restrictiveness of the mental health legislation in effect) need to contextualise these analyses.

Earlier meta-analytic work by Nielssen & Large^5^ suggested that approximately 40% of homicides committed by people with a psychotic illness occur during the first episode of psychosis and prior to treatment initiation. Results from our study suggested that a minority of homicides occurred in the context of a first episode of psychosis, since approximately three-quarters (75.7%) of the sample had prior (lifetime) contact with mental health services and a similar proportion (74.2%) had one or more in-patient admission preceding the index offence. Although this contact may have been for other clinical concerns unrelated to psychosis and/or risk of violence, they nevertheless represent potential missed opportunities to intervene more assertively and prevent harm – particularly for those contacts with services occurring in close temporal proximity to the homicide offence.

In this respect, we found that half (48.1%) of the individuals in our sample had a relationship with a mental health service provider in the year before the index homicide offence (including 28.4% with an in-patient admission), but that most of this contact was infrequent or sporadic. Unfortunately, the exact reason(s) as to why this contact may have been insufficient (e.g. in terms of frequency, intensity or consistency) could not be determined from the data available. A large proportion of this sample presented with ongoing substance use concerns, which may have contributed to a loss of continuity and/or adherence to care in the community prior to forensic tenure, and emphasises the need for multimodal interventions that can address the needs of those presenting with significant comorbid substance use.

Current findings align with prior studies conducted in samples of homicide offenders with schizophrenia (but not necessarily forensically dispositioned, i.e. NCRMD or UST) showing elevated rates of missed contacts with service providers in the time preceding the offence, as well as non-adherence to treatment plans.^15,18^ Further, despite the finding that many individuals in this sample had been ill for many years – on average, 8 years – prior to the commission of the index offence and that the majority had one or more prior psychiatric admissions, just 38.3% had a prescription for medication at the time of the homicide. Of this group, less than one-third were deemed to be sufficiently adherent. Golenkov et al,^38^ in their sample of 133 homicide offenders with schizophrenia in the Chuvash Republic of Russia, similarly observed many cases of missed appointments with treating agencies and non-adherence with antipsychotic medication in the period before the homicide.

### Patient and victim types

The current sample was comprised almost exclusively of young men with psychotic illness. Women comprise approximately 15% of the forensic patient population in Canada, but our sample contained just 7 women (6%). This reflects that one of our data collection sites was a male-only facility, but is also consistent with the finding that the most serious forms of violence still tend to be dominated by men. Individuals in this sample otherwise showed rates of personality and substance use disorders, as well as rates of prior criminal justice involvement, comparable to those for the larger and more heterogeneous population of forensic service users in Canada^39–41^ (see Supplementary Table 1, available at https://doi.org/10.1192/bjo.2023.567). Perhaps indicative of better premorbid adjustment, individuals in this sample were less likely to be homeless or without fixed address at the time of the offence and more likely to be living with family members. Individuals in the current sample were found to have offended significantly more frequently against family members or someone known to them (e.g. a roommate or neighbour), and less frequently against strangers, compared with these larger studies of Canadian forensic service users, which have included all offence types.

Current findings regarding victim type echo results from other jurisdictions, for example in an updated sample of Russian homicide offenders with schizophrenia^25^ (*n* = 179) where most victims were familial (57.0%) or known (39.5%) to the individual and where just 3.5% of victims were strangers. Similarly, in a representative sample of Belgian NGRI acquittees, those who had committed homicide or attempted homicide (*n* = 105) were more likely to have a family member victim (59.8%) and less likely to have a stranger victim (15.5%) compared with NGRI acquittees who had committed less severe violence (*n* = 571; 32.3 and 32.8% for family and stranger victims respectively).^26^ Family members are also injured more seriously than other types of victim,^40^ a finding that may reflect a higher threshold for reporting crimes within the family and stronger emotional ties resulting in more unrestrained violence.^42^

Persons with SMI who are in contact with the criminal justice system often have smaller social networks than the general population and those networks consist mainly of family members.^43^ Thus, family members – the majority of whom are parents – are more likely to be victimised because they have the most contact with their relative and are also more likely to be in a caregiving role.^26^ There may also be poor acceptance of mental illness among both patients and their family members, resulting in inadequate monitoring and treatment of symptoms.^44^ Providing better supports and psychoeducation to family members caring for a relative with SMI thus appears critical to manage risk, reduce rates of intra-familial violence and alleviate the isolation and emotional burden often felt by these caregivers.^45,46^

### Age at onset of symptoms and at first offence

Most patients in this sample first experienced symptoms of SMI prior to, or contemporaneously with, their first point of contact with the criminal justice system. This included those for whom admission to forensic care for the homicide offence was their first contact with both the mental health and criminal justice systems. In contrast, less than one-quarter of the sample had criminal justice contacts before symptom onset. The closest comparator study is of 109 Finnish persons with schizophrenia who were accused of homicide between 1983 and 2002,^30^ where similar proportions of early starters and late starters were observed. When compared with other studies of forensic patients in which varying proportions have committed homicide (Table 1), our percentage of early starters is relatively lower and our percentage of late starters relatively higher, as hypothesised. The proportion of late late starters in this sample is comparable with the two existing studies investigating this subgroup.^23,24^ In drawing these comparisons, it is also important to be mindful of the methods by which early-starter and late-starter groups were defined. Consistent with the method adopted in this study, Crocker et al,^20^ Jones et al,^47^ Simpson et al^23^ and van Dongen et al^24^ constructed subgroups using indicators of illness onset in relation to offending onset. In contrast, other studies^30–32^ have restricted their categorisation of early-starter and late-starter groups to the onset of behavioural problems (e.g. presence of conduct disorder, age at first conviction).

The finding of comparatively more late starters and late late starters in this sample suggests that many persons who are admitted to forensic care for homicide act primarily in the context of illness, with few other aggravating risk factors. This is supported by findings that individuals who commit homicide while actively unwell, compared with those without symptoms at the time, are less likely to have previous convictions for violence or histories of substance misuse.^15^ Forensic patients who have committed homicide, compared with patients committing other types of offence, are also found to have less severe psychopathology, fewer previous compulsory admissions and criminal convictions, and generally better premorbid adjustment in terms of employment and socioeconomic status.^48^ Because the management of risk in these cases will be largely focused on illness stabilisation, successful attenuation of symptoms will substantially and directly mitigate risk. In contrast, those who have been admitted to forensic care for homicide but who also have premorbid behavioural adjustment problems and legal contacts may have multiple areas of risk that are more complex to manage effectively (e.g. personality disorder, substance misuse and problems with housing, employment and relationships).

Application of an age at onset framework thus permits a greater clarification of risk management approaches that may need to be tailored according to the timing of illness *vis-à-vis* violence and criminality. A strategy targeting mental healthcare will probably be differentially effective for the sizeable proportion of late starters/late late starters who may otherwise be at increased risk for violence in the context of emerging psychotic symptoms. By contrast, multi-modal interventions that span illness-related and criminogenic needs will be required to address the more complex and long-standing risk issues present among the early-starter group.

### Limitations

Limitations of this study included the fact that this was not a population-based study but rather a purposive sampling of patients admitted to forensic care at one of three provincial forensic hospitals in Ontario. Unlike other studies,^36,49^ we do not have a good measure of the proportion of homicide offenders with SMI who receive a correctional (versus forensic) disposal in Ontario or Canada. This information would have better contextualised the frequency with which the ‘not criminally responsible on account of mental disorder’ (NCRMD) defence is successfully applied in cases of homicide involving an accused person with a psychotic illness. The generalisation of current results to other jurisdictions may therefore be limited and our comparison with existing population-based studies of forensic patients must be evaluated with this in mind. This was also a predominantly White male sample, with few females and other ethnicities represented. Questions remain as to whether gender and ethnicity affect the trajectory and motivations for serious violence in the context of SMI.

Data were missing for some of the historical variables examined (i.e. prior psychiatric admissions (available cases: *n* = 89) and criminal justice contacts (*n* = 82)) and the coding of frequency of contact with mental health services was based on information available in the health record. Further, sample sizes became small when partitioned by age at onset of illness and offending. We also note that some individuals in this sample had committed their index offence many years ago (one-third of the sample had their NCRMD finding 20 or more years ago), creating the possibility that the questions examined here in relation to service utilisation and age at onset show time-based effects.

### Implications

Many individuals who are admitted to forensic care for an offence of homicide have had inadequate mental healthcare leading up to this point, highlighting what might have been missed opportunities to prevent some episodes of violence through improved community mental healthcare. Effective management of violence risk conferred by mental illness should follow best practice standards in the delivery of evidence-based interventions generally, and targeted to commonly observed risk factors in this population (e.g. medication-assisted cognitive–behavioural therapy for substance use relapse prevention; evidence-based components of first-episode psychosis services such as community and family group psychoeducation, peer support and acute-phase care^50^; specialised and supported employment and vocation services). Interventions will need to be multi-modal in nature, encompassing psychological and pharmacological therapies, in order to effectively address both illness-related and criminogenic domains.

## Supporting information

Penney et al. supplementary material Penney et al. supplementary material

## Data Availability

The data that support the findings of this study are available from the corresponding author on reasonable request.
