# Supplementary material for: Homicide in the context of psychosis: analysis of prior service utilisation and age at onset of illness and violence
Source: BJPsych Open. 2023 Sep 19;9(5):e171. doi: 10.1192/bjo.2023.567 (PMC10594185; doi:10.1192/bjo.2023.567)
Supplement: Penney et al. supplementary material [file S2056472423005677sup001.docx]

**Table 1**

*Comparison of Patients who Committed Homicide to the Population of Canadian Forensic Service Users*

|  | Current sample | Crocker et al. (2015) | Penney et al. (2019) | Chaimowitz et al. (2022) |
| --- | --- | --- | --- | --- |
| Study description | 112 men and women found NCRMD for homicide in Ontario between 1972-2021 | Retrospective cohort study of 1800 adults found NCRMD in British Columbia (*n* = 222), Quebec (*n* = 1094), and Ontario (*n* = 484) between 2000-2005 | All forensic admissions in Ontario from 1987-2012 resulting in a disposition of NCRMD (*N* = 2533) | Archival study of 1240 adults subject to the jurisdiction of the forensic system in Ontario between 2014-2015 (91.6 % NCRMD, 8.4% UST) |
| Age at index offense | 32.63 (11.99) _a_ | 36.56 (12.42) _b_ | 35.60 (12.29) _c_ | 32.00 (median) |
| Sex (% male) | 105 (93.8) _a_ | 1519 (84.4) _b_ | 2171 (85.7) _b_ | 1063 (85.7) _b_ |
| Residential status (% no fixed address) | 3 (2.8) _a_ | 144 (9.2) _b_ | 374 (16.7) _c_ | Not reported |
| % primary psychotic disorder | 106 (94.5) _a_ | 1268 (70.9; at verdict) _b_ | 2076 (82.0) _c_ | 1012 (81.6) _c_ |
| % substance use disorder | 56 (50.0) _a,c_ | 550 (30.8; at verdict) _b_ | 1230 (48.6) _a_ | 709 (57.2) _c_ |
| % personality disorder | 32 (28.6) _a_ | 190 (10.6; at verdict) _b_ | 762 (30.1) _a_ | 345 (27.8) _a_ |
| % with prior psychiatric admissions | 66 (74.2) _a_ | 1051 (72.0) _a_ | 1420 (61.1) _b_ | 1016 (83.1) _c_ |
| % with prior convictions | 31 (48.4) _a,b,c_ | 838 (46.6) _a_ | 1377 (56.3) _b_ | 709 (57.2) _b,c_ |
| Victim type |  |  |  |  |
| Family member | 62 (55.4) _a_ | 365 (33.7) _b_ | 588 (25.5) _c_ | 252 (24.3) _c_ |
| Friend / acquaintance | 20 (17.9) _a,b_ | 143 (13.2) _a_ | 311 (13.5) _a_ | 163 (16.5) _b_ |
| Neighbor / roommate | 20 (17.9) _a_ | 44 (4.1) _b_ | 263 (11.4) _c_ | 29 (2.9) _b_ |
| Stranger | 10 (8.9) _a_ | 246 (22.7) _b_ | 664 (28.8) _c_ | 328 (33.2) _c_ |

*Note*. Values in the same row that do not share subscripts differ at *p* ≤ .05. NCRMD = Not Criminally Responsible on account of Mental Illness. UST = Unfit to Stand Trial. Percentages account for missing data, and represent the valid percent using all cases available.
